# Regulation of chemokine and chemokine receptor expression by PPARG in adipocytes and macrophages

**DOI:** 10.1186/1479-5876-9-S2-P23

**Published:** 2011-11-23

**Authors:** MT Audrey Nguyen, Ai Chen, Wendell J Lu, WuQiang Fan, Ping-Ping Li, Dayoung Oh, David Patsouris

**Affiliations:** 1 Dept. of Medicine, University of California, San Diego, La Jolla, USA; 2 Laboratoire CarMeN, INSERM U1060, Faculté de médecine Lyon Sud, Oullins, France

## Background

PPARG plays a key role in adipocyte biology, and Rosiglitazone (Rosi), a thiazolidinedione (TZD)/ PPARG agonist, is a potent insulin-sensitizing agent [1-3]. Recent evidences demonstrate that adipose tissue inflammation links obesity with insulin resistance and that the insulin-sensitizing effects of TZDs result, in part, from their anti-inflammatory properties [4,5]. However the underlying mechanisms are unclear. Free Fatty Acids (FFAs) are important adipocyte-derived signaling molecules whose plasma levels are elevated in obese and insulin resistant individuals and animal models [6,7]. In this study, we establish a link between free fatty acids (FFAs) and PPARG in the context of obesity-associated inflammation.

## Methodology and methods

We used 3T3L1 mouse cells as a model of mature adipocytes. Conditioned media were prepared from 3T3L1 mature adipocytes exposed to different conditions and subsequently used for *in vitro* chemotaxis assays with Raw264.7 mouse macrophages cells. 10 weeks old males C57Bl6 mice (littermates) were fed a high fat diet (60% Kcal fat, Research Diet) where rosiglitazone was directly mixed by manufacturer. Normal chow diet consisted of 13.5% kcal fat (Lab Diet).

## Results

We show that treatment of adipocytes with FFAs downregulates PPARG protein and mRNA levels. Knockdown of adipocyte PPARG resulted in upregulation of MCP1 gene expression and secretion, leading to enhanced macrophage chemotaxis. Rosi inhibited these effects. In a high fat feeding mouse model, we show that Rosi treatment decreases recruitment of proinflammatory macrophages to epididymal fat. This correlates with decreased chemokine and decreased chemokine receptor expression in adipocytes and macrophages, respectively.

## Conclusions

In summary, we describe a novel link between FAs, PPARG, adipocytes, and adipocyte-driven recruitment of macrophages and thus provide an additional potential mechanism for the anti-inflammatory and insulin-sensitizing actions of TZDs.
